# Development of Low Parasitic Light Sensitivity and Low Dark Current 2.8 μm Global Shutter Pixel †

**DOI:** 10.3390/s18020349

**Published:** 2018-01-25

**Authors:** Toshifumi Yokoyama, Masafumi Tsutsui, Masakatsu Suzuki, Yoshiaki Nishi, Ikuo Mizuno, Assaf Lahav

**Affiliations:** 1 TowerJazz Panasonic Semiconductor Co., Ltd., 800 Higashiyama, Uozu City, Toyama 937-8585, Japan; tsutsui.masafumi@tpsemico.com (M.T.); suzuki.masakatsu@tpsemico.com (M.S.); nishi.yoshiaki@tpsemico.com (Y.N.); mizuno.ikuo@tpsemico.com (I.M.); 2 Tower Semiconductors, Migdal Haemeq 23105, Israel; asafla@towersemi.com

**Keywords:** global shutter, parasitic light sensitivity (PLS), dark current

## Abstract

We developed a low parasitic light sensitivity (PLS) and low dark current 2.8 μm global shutter pixel. We propose a new inner lens design concept to realize both low PLS and high quantum efficiency (QE). 1/PLS is 7700 and QE is 62% at a wavelength of 530 nm. We also propose a new storage-gate based memory node for low dark current. P-type implants and negative gate biasing are introduced to suppress dark current at the surface of the memory node. This memory node structure shows the world smallest dark current of 9.5 e^−^/s at 60 °C.

## 1. Introduction

CMOS image sensors with global shutter (GS) are becoming popular [1,2,3]. It is possible to capture the shape of a high speed moving object with high accuracy without distortion. Conventional CMOS sensors adopt rolling shutter (RS) method. In the RS method, an exposure is sequentially performed for each row pixel and there is a slight time difference in signal readout for each row pixel. Thus, the high-speed moving object is distorted. For example, when a flash is used during shooting, the flash band phenomenon may occur with different brightness of the image on the top and the bottom. CMOS image sensor with GS exposes all the pixels at the same time and it can take a non-distorted photograph of the high speed moving object such as a rotating propeller. Therefore, it is expected to be utilized in the automotive field and industrial applications such as inspection cameras.

GS CMOS image sensor with small pixel is demanded for high resolution pictures and sensing. In practical use of GS sensor, it is important to achieve high signal-to-noise ratio. High quantum efficiency (QE) is required to increase the signal intensity. In order to increase the QE, it is necessary to provide more light to the photo diode (PD) even in small size pixel. In terms of noise, it is necessary to care the noise caused by GS structure. 

**Figure 1:**
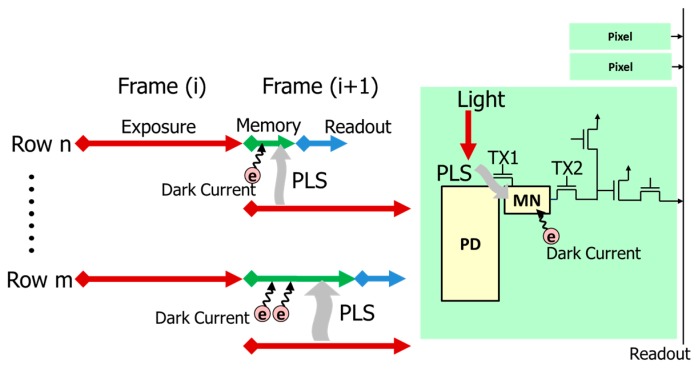
Configuration and reading method of GS pixel.

Figure 1 shows a simple configuration and reading method of GS pixel. In order to realize GS capability, a memory node (MN) must be added in each pixel [4]. The electrons stored in the PD are collectively read out to MN and used as an image signal. The exposure is performed at once, but the readout is performed for each row as shown in Figure 1. Therefore, the electrons are generated in the MN before the readout becomes noise, and leads to deterioration in image quality. When an image is output with much noise, the lower part of the picture becomes brighter or noisy because the noise is increased in later read rows. Accordingly, it is necessary to reduce the noise generated in the MN. Major causes of the noise are light penetration into MN and dark current.

PLS is generated by light incident into MN. An increase of PLS leads to deterioration of image quality. This is because the charges generated by incident light to MN are added to the charges stored in MN after the exposure. In this paper, we present the advanced approach of optical design in 2.8 μm GS pixel for drastic improvement of PLS and QE [5]. We expand our original paper with a new storage-gate based MN for low dark current.

**Figure 2:**
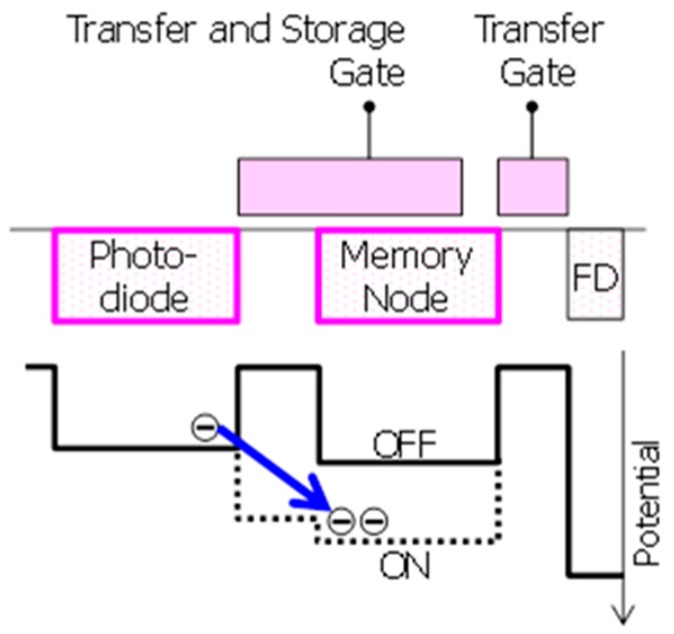
Schematic and potential diagram of storage-gate based memory node.

Figure 2 shows a schematic and potential diagram of storage-gate based MN. For small GS pixel, dual transfer GS pixels are suitable from the point of views of scalability, low read noise, and low dark current. Their fewer components in pixels offer a better scalability compared to 8T voltage domain pixels [6].

In dual transfer GS pixels, the photo-generated charges are transferred to MN. This allows a correlated double sampling (CDS) on a floating diffusion in successive transfer, which can reduce pixel noise to below 1 to 2 electrons [7,8]. This read noise is much better than that of 5T charge transfer pixels without CDS [1]. Additionally, such MN shows much smaller dark current compared to floating diffusion used as MN in 5T-GS pixels [9]. Considering the MN in dual transfer GS pixels, pinned MN and storage-gate based MN were reported [8,9]. In the storage-gate based MN shown in Figure 2, the storage-gate can control the depletion potential of the MN. This offers better charge transfer from the PD to the MN. Furthermore, using a single poly-Si gate on MN for both transfer and storage can enlarge its gate area, and it can enhance potential controllability. It makes the storage-gate based MN suitable for small GS pixels. Although the reported dark current of storage-gate based MN is higher than that of the pinned MN due to dark current generation at the surface of MN [8], its potential controllability can also improve dark current in combination with negative gate biasing.

This paper reports on newly developed storage-gate based MN structures to suppress dark current with negative gate biasing and surface p-type implants. The generation position of dark current identified by the test structures is also described.

Section 2 describes the device structure. Section 3 discusses reduction of PLS, and Section 4 discusses the reduction of dark current. Conclusions are presented in Section 5.

## 2. Device Structure

**Figure 3:**
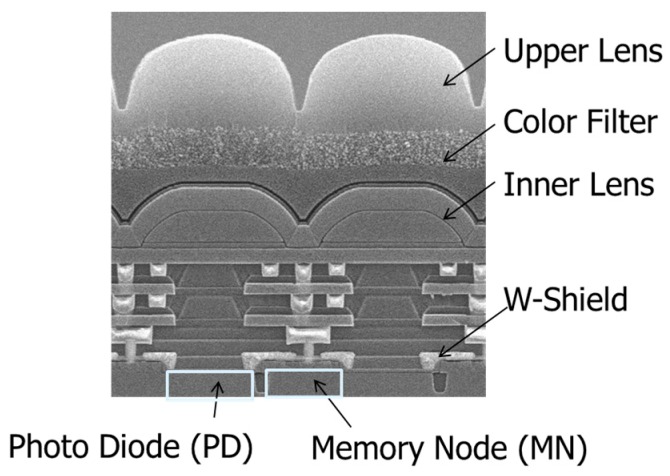
Cross-section of GS pixel.

Figure 3 shows the cross-section of global shutter pixel structure we developed. The pixel was designed with 110 nm node process, including double micro lens, three-layer Cu wiring and tungsten (W) light shield structures. The incident light is collected by the upper lens and the inner lens, then passes between the Cu-wirings, and finally enters the PD. The MN is placed next to the PD. W light shield is placed just above the MN to block an incident light as shown in Figure 3 [10,11].

**Figure 4:**
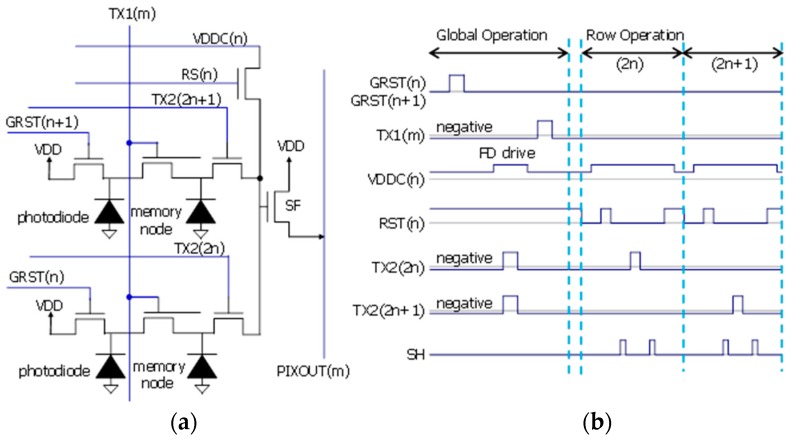
Pixel structure. (**a**) Pixel circuit schematic; (**b**) Timing diagram.

Figure 4a shows a schematic diagram of pixel circuit and Figure 4b shows a timing diagram. In order to increase the PD area and MN area, we use a 2 × 1 sharing scheme. The pixel has no row-select and the floating diffusion drive method is used [12]. TX1 is served as a control line for both a transfer gate from the PD to MN and a storage-gate over MN to reduce a control line for the storage-gate. During operation, except for transferring electron from PD to MN, TX1 and TX2 biases are kept negative for suppressing dark current, where accumulated holes at the surface occupy recombination centers and prevent carrier generation [9].

## 3. Reduction of PLS

### 3.1. Structural Issue of Small GS Pixels in PLS Reduction

**Figure 5:**
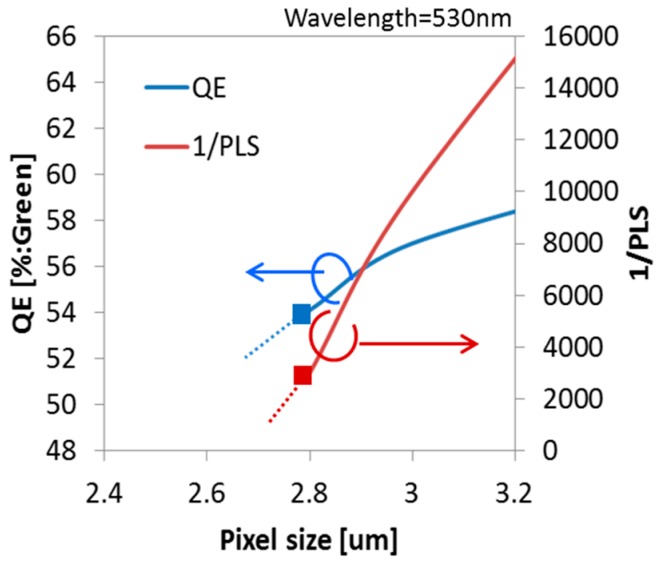
Pixel size dependence of QE and 1/PLS.

In order to reduce PLS, it is necessary to reduce the incident light to MN. Figure 5 shows the pixel size dependence of QE and 1/PLS. These data were estimated by optical simulations. Simulation was carried out at the green pixel (wavelength = 530 nm). As the pixel size becomes smaller, QE and 1/PLS are seriously degraded. The degradation of these optical characteristics is due to the fact that the incident light is not concentrated efficiently in the optical aperture. It is not easy to change the wiring width and height in order to keep wiring resistance and capacitance even if the pixel size is scaled down. Therefore, the optical aperture size becomes smaller and it becomes difficult to pass the light between Cu-wirings in small pixels. In this paper, we present the advanced approach of optical design in 2.8 μm GS pixel for drastic improvement of PLS and QE.

It is conceivable to expand the W light shield to prevent light incident on the MN. However, the QE decreases when the W light shield is expanded. It is difficult to realize both high QE and high 1/PLS by simply adjusting the W light shield extension. It is important to improve the optical characteristics by changing the propagation of light.

### 3.2. Effect of Double Micro Lens Structure

A double micro lens structure is often used as a method for efficient light collection into a narrow optical aperture [13,14]. An inner lens is placed above the Cu-wiring layers as shown in Figure 3. In the case of double lens structure, it is possible to adjust the light collection at two positions of the upper lens and the inner lens. It is expected that the light collected by the upper lens can be efficiently collected into the optical aperture. 

**Figure 6:**
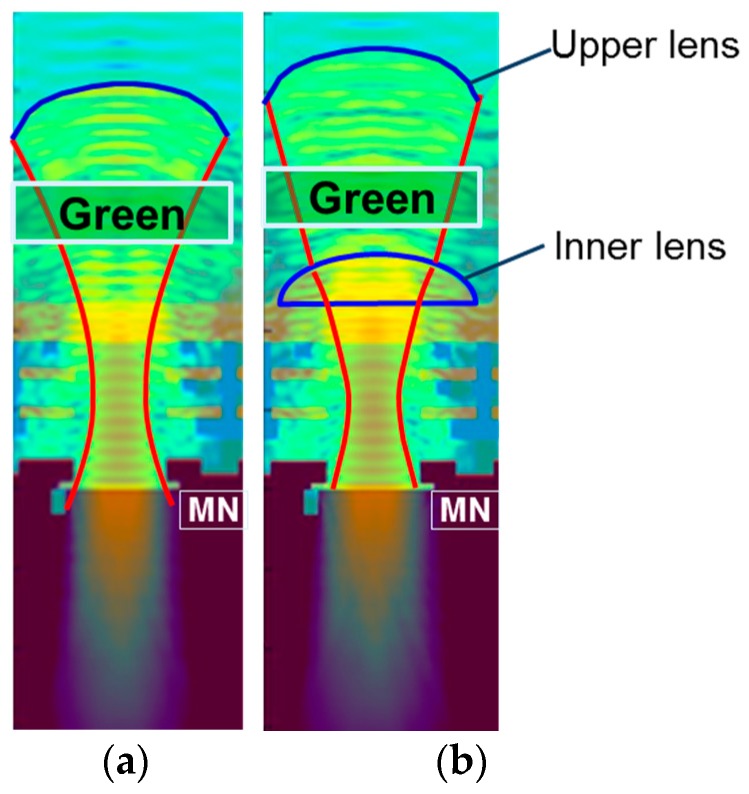
Comparison of lens structure. (**a**) Single lens; (**b**) Double lens. Green pixel, Wavelength = 530 nm.

We compared PLS and QE between with and without inner lens structures by optical simulation using three-dimensional finite difference time domain (FDTD) method. Figure 6 shows the electric field distribution, and Table 1 shows the simulation results. By adopting inner lens, the incident light was concentrated efficiently in the optical aperture as shown in Figure 6b. As a result, QE was improved from 54.1% to 58.9%, and 1/PLS was improved by 30%. From the above results, we decided to adopt the double lens structure. For further improvement, we studied the inner lens structure in more detail.

### 3.3. Analysis and Design Concept of Inner Lens (Our Proposal)

**Figure 7:**
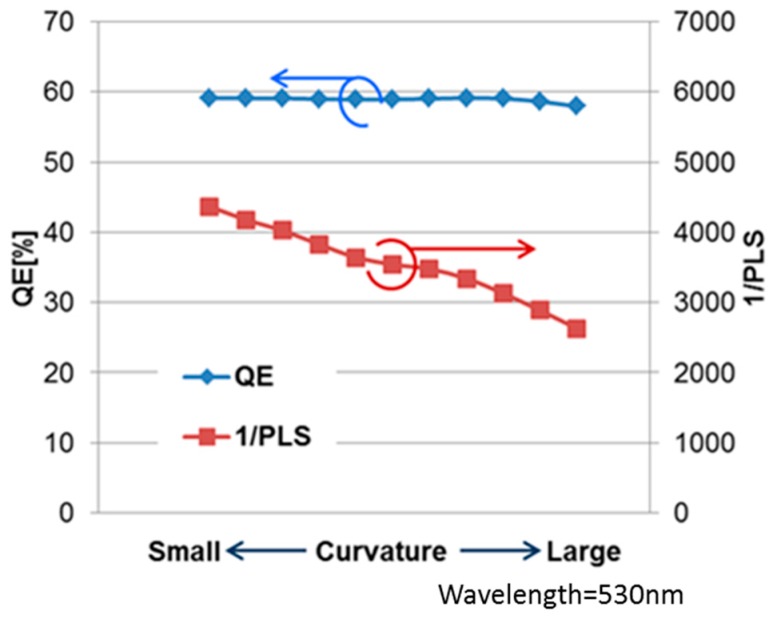
Curvature dependence of QE and 1/PLS.

**Figure 8:**
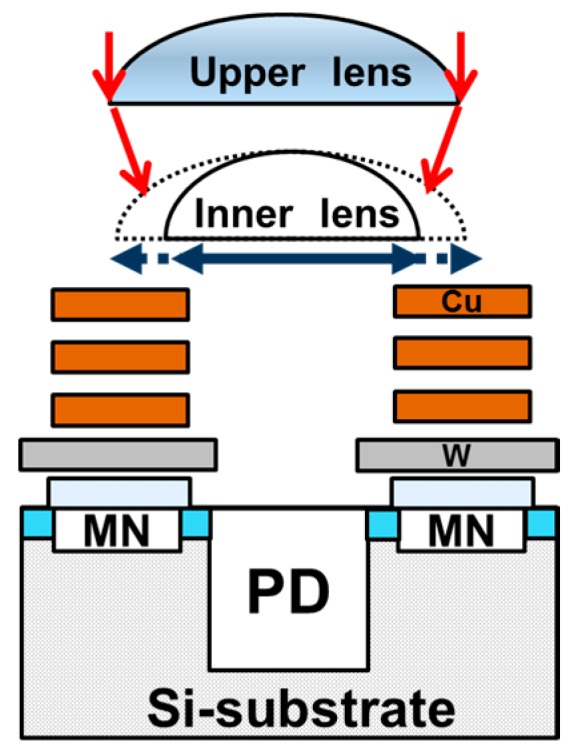
Simulation condition.

We analyzed the inner lens curvature dependence of QE and PLS by optical simulation. Figure 7 shows the simulation results. These data were calculated by changing a diameter of inner lens (constant lens height) as shown in Figure 8. 

PLS depends on curvature, and small curvature was better for PLS. On the other hand, QE was stable in the curvature range. It indicates that once the incident light collected by the upper lens enters the inner lens, the light reaches the PD regardless of the inner lens curvature. Therefore, the design concept of the upper lens is to concentrate the light on the inner lens. On the other hand, 1/PLS depends on curvature, and small curvature was better for 1/PLS.

**Figure 9:**
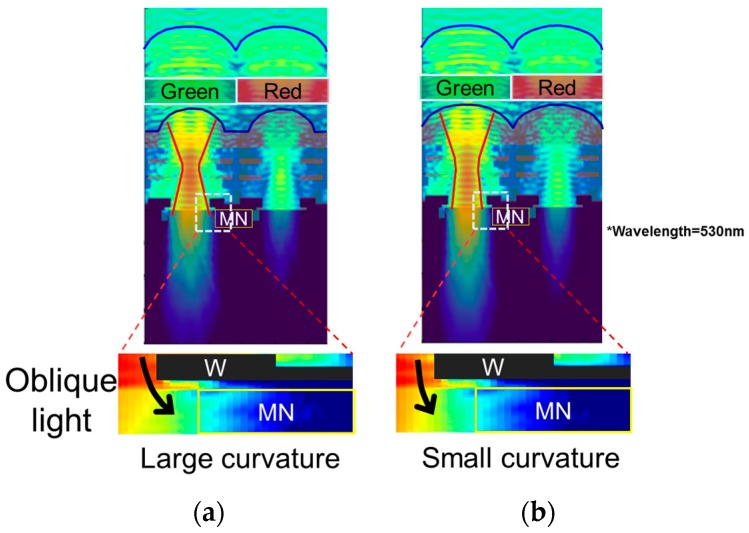
Cross-sections of electric fields. (**a**) Large curvature; (**b**) small curvature.

We compared the electric field between large and small curvature to analyze the key factor of PLS. Figure 9a,b shows the cross-section of electric field distribution. When the inner lens has a large curvature, the beam widely spreads near the W light shield as shown in Figure 9a. In the case of small curvature lens, the beam spread is narrower than that of large curvature lens. Then the beam enters almost perpendicularly to Si as shown in Figure 9b. 

We analyzed the electric field distribution near the MN in more detail. The bottom views of Figure 9a,b are the enlarged views of the broken line portion of upper figures. In the case of large curvature inner lens, a large amount of oblique light bent by the inner lens enters the MN as shown in Figure 9a. On the other hand, in the case of small curvature lens, the light entering the MN decreases as shown in Figure 9b.

These analyses show it is important to make the light incident perpendicular to Si in order to reduce oblique incidence to the MN. To summarize the results, the double lens design concept is as follows. The incident light collected by the upper lens should be concentrated on the inner lens in order to improve the QE, and the inner lens should be designed so that light enters straight into the Si.

**Figure 10:**
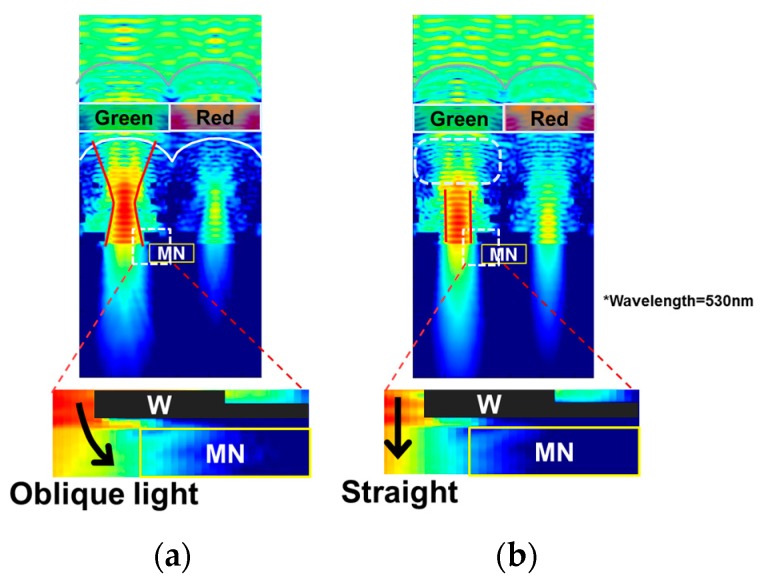
Comparison of the cross-sections of electric fields. (**a**) Conventional inner lens; (**b**) newly developed inner lens.

We developed the new inner lens based on this concept. Figure 10 shows the comparison of electric fields between conventional and newly developed inner lenses. In the case of the newly developed inner lens, we can see that the light enters straight into the silicon as shown in Figure 10b. The incident light to the MN is greatly reduced. Therefore, the simulated value of 1/PLS is greatly improved and is expected to be 7100.

### 3.4. Results of Newly Developed Inner Lens Based on Our Design Concept for Low PLS

**Figure 11:**
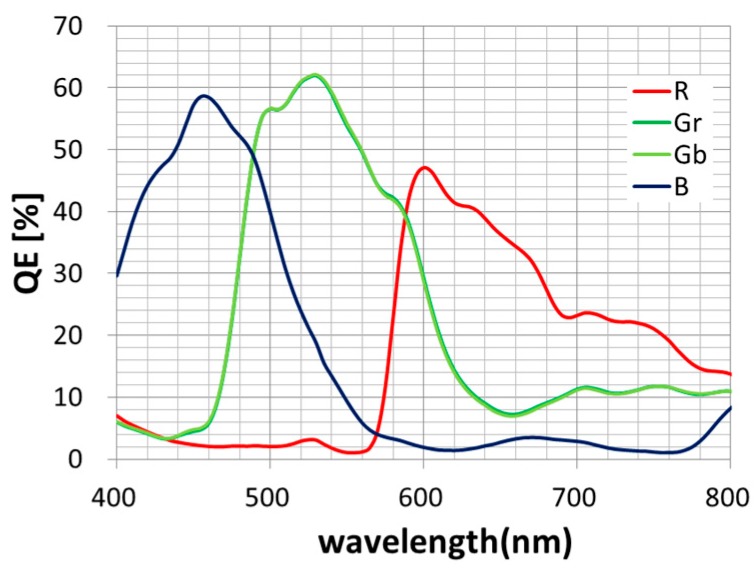
QE curves of 2.8 μm GS image sensor with newly developed lens.

We fabricated two types of 2.8 μm GS devices with conventional and newly developed inner lenses. Table 2 shows the measurement results. We confirmed the superiority of the new inner lens based on our concept. The simulation results agreed with the experimental results. The QE of green pixel is the same—62%—in both types. The inner lens developed with the new concept obtained two times better 1/PLS than a conventional lens and the value is 7700. The 1/PLSs of blue and red pixels are 3600 and 2600 (wavelength = 450 nm and 600 nm), respectively. Figure 11 shows the QE curves with the newly developed inner lens. The QE of blue and red pixels are 59% and 48%, respectively.

## 4. Reduction of Dark Current

### 4.1. Development of Low Noise MN Structures

**Figure 12:**
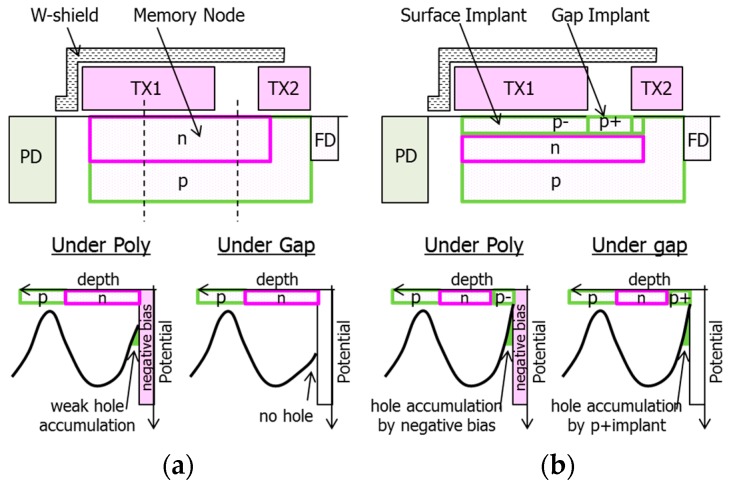
Schematic structure (**top**) and potential diagram (**bottom**) of (**a**) conventional MN and (**b**) proposed MN. Potential diagrams are shown along dotted lines in (a).

Figure 12a shows a conventional MN structure without p-type implants and its schematic potential diagram, and Figure 12b shows a proposed MN. In the conventional MN, the negative gate biasing can only accumulate holes weakly under poly-Si gates due to an n-type layer in the MN within the range of the negative voltage allowed by CIS operation. 

This causes larger TX1 gate voltage (VTX1) dependence of accumulated hole concentration and results in higher VTX1 dependence of dark current. The n-type layer in the MN also prevents the formation of the hole accumulation layer under a gap between TX1 and TX2. These two are the major factors of dark current generation in the conventional MN because the surface recombination center of the gate oxide under poly-Si is not filled by holes.

The proposed MN has p-type implants both under the poly-Si gates and under the gap in order to accumulate holes at the surface with negative gate biasing. The surface p-type implant makes a threshold voltage of the MN higher and makes hole accumulation better with the negative gate biasing, and then makes VTX1 dependence of dark current lower. High p-type concentration of the gap p-type implant is required to accumulate holes under the gap because of the lower effectiveness of negative gate biasing in the region under the gap. Higher p-type concentration under the gap makes electrostatic potential in n-type layer under the gap higher, and makes it difficult for electrons to transfer from MN to FD. Maximum p-type dosage for the gap implant should be determined considering a full well capacity and image lag.

**Figure 13:**
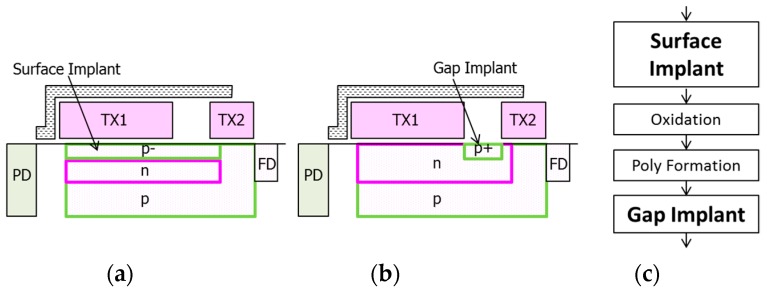
Test structure of the memory node. (**a**) Surface implant only; (**b**) gap implant only; (**c**) process flow.

For evaluating the dark current of the proposed MN, we examined three MN structures for 2.8 μm GS pixels. One is the proposed MN, another is the MN with only the surface-implant illustrated in Figure 13a, and the other is the MN with only the gap-implant illustrated in Figure 13b. The two structures illustrated in Figure 13 are test structures to identify generation positions of dark current. 

Process flow is shown in Figure 13c. The gap p-type layer was formed by implantation in a self-aligned manner using TX1 and TX2 poly-Si gates. Low thermal budget after the gap implant results in shallow p-type profile. This allows usage of higher dosage for the gap-implant as required.

**Figure 14:**
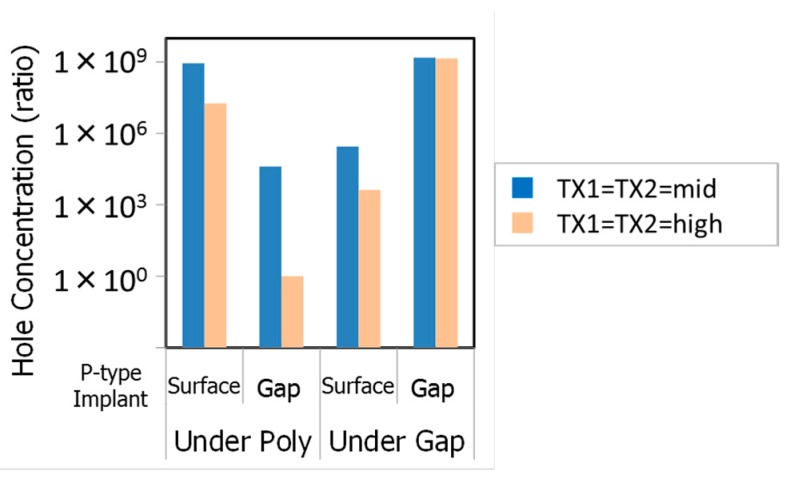
P-type implants and VTX1 dependencies of hole concentration at the surface of Si.

All three kinds of MNs were designed to satisfy the target of the full well capacity and the image lag at the MN. Simulation results of relative hole concentration at the surface under the poly-Si gate and under the gap in the test structures are shown in Figure 14. The gap-implanted MN has a higher hole concentration under the gap than that of the surface-implanted MN as expected. Low VTX1 and VTX2 dependence of hole concentration of the gap-implanted MN under the gap also indicates well-accumulated hole region under the gap in this MN. The hole concentration under the poly-Si gate in the surface-implanted MN shows smaller VTX1 dependence and higher hole concentration than those of the gap-implanted MN. It is expected to be smaller VTX1 dependence of dark current in the surface-implanted MN.

### 4.2. Experimental Results of Dark Current

**Figure 15:**
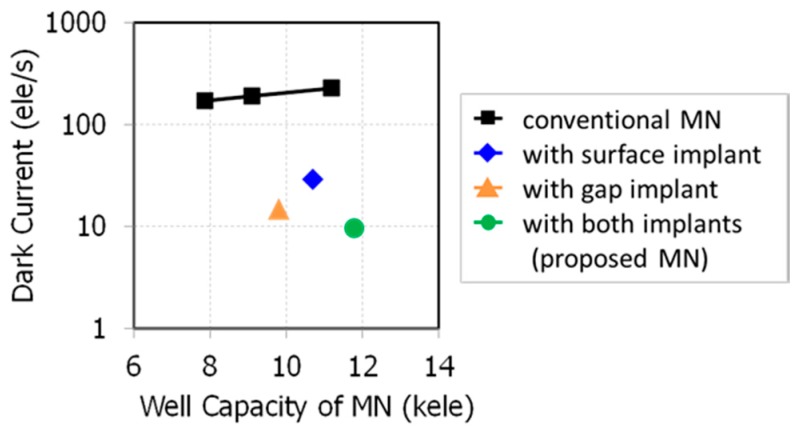
Relationship between well capacity and dark current of MN at 60 °C.

Figure 15 shows the measured dark currents of developed MNs. The dark currents of our developed MNs are suppressed to 1/10 at 60 °C, compared to the conventional MN without p-type implants when the full well capacities of the MNs are the same. The shown full well capacity is defined as signal without transferring electrons from PD to MN under over-saturated light and without W-shield for measurement. In this condition, PLS and blooming fill MN with electrons to its maximum signal level. The dark currents of the surface-implanted MN, the gap-implanted MN, and the proposed MN at VTX1 = mid are 24.1 e^−^/s, 12.9 e^−^/s, and 9.5 e^−^/s, respectively. 

**Figure 16:**
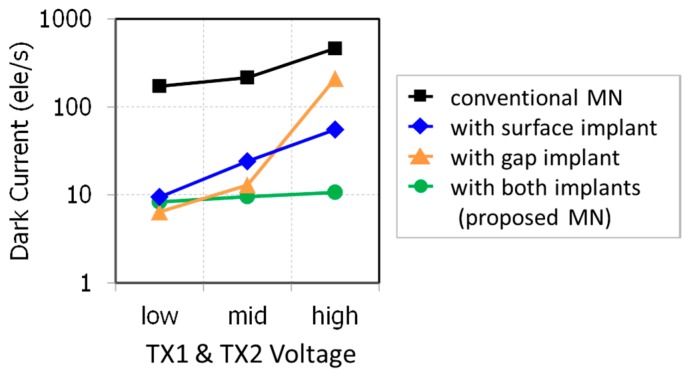
VTX1 dependencies of dark current of MN at 60 °C.

Figure 16 shows VTX1 and VTX2 dependence of dark current of each MN. Thanks to p-type implants, the dark currents of the newly developed MNs are smaller than that of the conventional MN in the measured range of VTX1. The high dark current of the gap-implanted MN at VTX1 is higher than that of the surface-implanted MN. Comparing calculated hole concentration under the poly-Si gate shown in Figure 14, this difference of the dark current comes from the surface under the poly-Si gate. 

This indicates that negative bias is not enough for suppressing the gate voltage dependence of dark current and the p-type surface implant is required to stabilize the dark current in the storage-gated MN as expected. At VTX1 = mid, the dark current of the gap-implanted MN is smaller than that of the surface implanted MN. This is because after accumulating holes enough under the poly-Si gate with low VTX1, the carrier generation under the gap dominates dark current.

**Figure 17:**
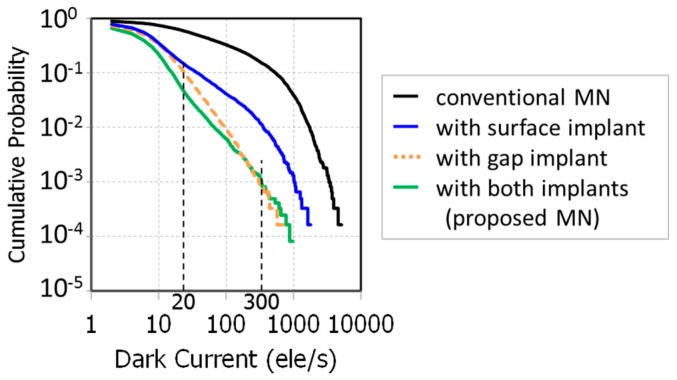
Cumulative probabilities of dark current for the developed memory nodes with VTX1 and VTX2 = mid at 60 °C.

**Figure 18:**
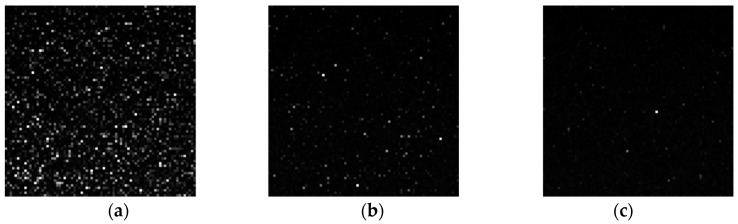
Dark images at 60 °C. (**a**) Conventional; (**b**) MN with surface implant; (**c**) MN with surface and gap implant. Analog gain ×4, digital gain ×32, Tint_pd = 100 ms, Tint_mn (max) = 33 ms, 24 frames averaged.

Figure 17 shows cumulative probabilities of dark current at VTX1 = mid. The proposed MN and the gap-implanted MN have similar distribution. On the other hand, the surface-implanted MN has a higher probability of dark current over 20 e^−^/s. Considering the difference of implanted p-type layers, these outliers come from the surface under the gap. Figure 18 shows the dark images at 60 °C. White spots caused by dark current were drastically reduced in the proposed MN as shown in Figure 18c. Table 3 contains a list of pixel performance of the newly developed 2.8 μm GS pixel. Our developed MN structure shows the world smallest dark current of 9.5 e^−^/s at 60 °C.

## 5. Conclusions

We developed a low PLS and low dark current 2.8 μm GS pixel. We proposed a new lens design concept for low PLS. The double lens structure was adopted. The double lens design concept we proposed is as follows:

(1) The incident light collected by the upper lens should be concentrated on the inner lens in order to improve the QE.
(2) The inner lens should be designed so that light enters straight into the Si.

We developed a new inner lens based on the proposed concept. We achieved both a high QE of 62% and a high 1/PLS of 7700 in 2.8 μm GS pixel. 

We also proposed and developed new MN structures for low dark current. We verified that high hole concentration under the gap between TX1 and TX2 poly-Si gates was required to suppress MN dark current. Our developed MN structure shows the world smallest dark current of 9.5 e^−^/s at 60 °C.

## Figures and Tables

**Table 1:** Results of optical simulations

	Single Lens	Double Lens
QE (%)	54.1	58.9
1/PLS	2700	3500

Green pixel, Wavelength = 530 nm.

**Table 2:** Measurement results of optical characteristics.

		Without Inner Lens	Conventional Inner Lens	Newly Developed Lens for GS
QE (%)	simulation	54.1	58.9	58.6
	measurement		62.0	62.0
1/PLS	simulation	2700	3500	7100
	measurement		4000	7700

Green pixel, Wavelength = 530 nm.

**Table 3:** Pixel performance of developed 2.8 μm GS pixel.

	This Work	Ref. [10]	Unit
Process node	110 nm	-	-
Pixel pitch	2.8	2.8	μm
Linear Qsat	7	6	ke^−^
Dark current @ PD	14	-	e^−^/s
Dark current @ MN	9.5	60	e^−^/s
